# Pre‐meiotic deletion of PEX5 causes spermatogenesis failure and infertility in mice

**DOI:** 10.1111/cpr.13365

**Published:** 2022-11-26

**Authors:** Min Liu, Shuangyuan Liu, Chenyang Song, Haixia Zhu, Bin Wu, Aizhen Zhang, Hui Zhao, Zongzhuang Wen, Jiangang Gao

**Affiliations:** ^1^ Medical Science and Technology Innovation Center Shandong First Medical University Jinan China; ^2^ School of Life Science and Key Laboratory of the Ministry of Education for Experimental Teratology Shandong University Jinan China; ^3^ Department of Reproductive Medicine, Jinan Central Hospital, Cheeloo College of Medicine Shandong University Jinan China

## Abstract

Peroxisomes are involved in the regulation of various pathological processes. Peroxisomal biogenesis factor 5 (PEX5), which plays an essential role in peroxisomal biogenesis, is critical for reactive oxygen species (ROS) accumulation. However, its underlying functions in spermatogenesis have not yet been identified. *Pex5* was deleted by crossing *Stra8‐Cre* mice with *Pex5* ^*flox/flox*^ mice before the onset of meiosis. The morphology of testes and epididymides, spermatogenesis function, and fertility in both wild type (WT) and *Pex5* ^−/−^ mice were analysed by haematoxylin and eosin (HE) and immunofluorescent staining. Mechanism of PEX5 affecting peroxisomes and spermatogenesis were validated by Western blot and transmission electron microscopy (TEM). Transcriptome RNA sequencing (RNA‐seq) was used to profile the dysregulated genes in testes from WT and *Pex5* ^−/−^ mice on postnatal day (P) 35. The adult *Pex5* knockout male mice were completely sterile with no mature sperm production. Loss of *Pex5* in spermatocytes resulted in multinucleated giant cell formation, meiotic arrest, abnormal tubulin expression, and deformed acrosome formation. Furthermore, *Pex5* deletion led to delayed DNA double‐strand break repair and improper crossover at the pachytene stage. Impaired peroxisome function in *Pex5* knockout mice induced ROS redundancy, which in turn led to an increase in germ cell apoptosis and a decline in autophagy. *Pex5* regulates ROS during meiosis and is essential for spermatogenesis and male fertility in mice.

## Graphical abstract

We discovered the *Pex5* participated in the regulation of spermatogenesis. *Pex5* knockout aggravated ROS imbalance. Mechanistically, *Pex5* deletion led to delayed DNA double‐strand break repair and homologous recombination. Impaired peroxisome function in *Pex5* knockout mice induced ROS redundancy, which in turn led to an increase in germ cell apoptosis and a decline in autophagy.

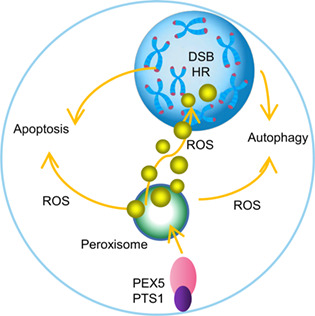

## 1 INTRODUCTION

The peroxisome is a cellular rheostat of reactive oxygen species (ROS). Peroxisomes can quench endogenous and exogenous ROS.^1^, ^2^ Peroxisomal biogenesis factor 5 (PEX5), a predominant receptor for the transport of peroxisomal matrix proteins to modulate redox homeostasis,^3^ is an essential component of peroxisomes involved in peroxisomal protein import by recognizing peroxisomal targeting signal 1 (PTS1).^4^ Catalase (CAT) is one of the abundant ROS‐scavenging enzymes, which are delivered into the peroxisome by PEX5, protecting cells from ROS‐induced stress. CAT knockdown potentiates ROS‐mediated apoptosis in Vor‐sensitive cells,^5^ whereas PEX5 knockdown inhibits the import of CAT into peroxisomes and augments cellular ROS accumulation.^6^ Under normal physiological conditions, ROS oxidizes cellular nucleotide pools and causes DNA double‐strand breaks (DSBs), and DNA repair may occur downstream.^7^, ^8^ If DSBs are neither repaired nor removed, the DNA damage response triggers cell death.^9^, ^10^ On radiation exposure, PEX5 participated in DSBs repair and homologous recombination by consuming excessive ROS,^11^ which protected hepatocellular carcinoma cells from radiation‐induced damage.

Spermatogenesis is a complex and dynamic process that involves the proliferation and differentiation of spermatogonia, meiotic division of spermatocytes, and morphological transformation of round spermatids in the seminiferous tubules of the testes^12^ Meiotic failure is the leading cause of sterility and birth defects.^13^, ^14^ The main steps of meiosis are DNA DSBs repair and crossover recombination.^15^, ^16^ These steps not only establish the physical connections between homologous chromosomes but also promote the exchange of genetic information between parents required for proper chromosome segregation^17^ Some proteins play critical roles in DNA DSBs repair and crossover recombination. For example, *Meiok21* knockout greatly disrupts DSB repair, synapsis, and crossover formation in spermatogenesis in mice.^18^ Prohibitin promotes meiotic DSB repair and homologous recombination both in vitro and in vivo.^19^ *Cxxc1*‐deleted spermatocytes fail to complete meiosis and are arrested at the secondary spermatocyte stage.^20^ Given the potential functions of PEX5 in clearing ROS and DNA DSBs repair, it is worth investigating whether PEX5 is also required for the early stages of meiosis and regulating spermatogenesis in mice. In addition, PEX5 is an autophagy‐related peroxisomal protein,^21^ and defects in PEX5 can lead to apoptosis.^1^ Autophagy and apoptosis play important roles in testicular injury and spermatogenesis dysfunction.^22^, ^23^ However, evidence supporting the role of PEX5 in the reproductive system of male mice is lacking.

To determine the functional role of PEX5, a conditional *Pex5* knockout strain was generated to specifically ablate *Pex5* in pre‐meiotic germ cells using *Stra8‐Cre* mice. *Pex5* deletion caused an increase in ROS and apoptosis but a decrease in autophagy in the testes. DNA DSBs repair and homologous recombination were impaired at the pachytene stage of meiotic prophase. Some *Pex5*‐deleted spermatocytes failed to complete meiosis, leading to spermatogenesis failure and complete male infertility.

## 2 MATERIALS AND METHODS

### 2.1 Animals

All animal experimental protocols were approved by the Ethics Committee of Shandong First Medical University (W202111230331, Jinan, China). Mice care and use were strictly in accordance with Chinese animal protection laws.

*Pex5* ^*flox/flox*^ mice were generated using CRISPR/Cas9 technology and targeted homologous recombination (Cyagen, China). Two single guide Ribonucleic acids (sgRNAs) were designed for targeting introns two and three, sgRNA1: GGCCCTTGTGGAGCCGTCGTGGG, and sgRNA2: TGTCTGTGTAAAGCCGCAGAAGG. The two sgRNAs, Cas9 mRNA, and the donor vector containing loxP sites and homology arms were co‐injected into fertilized mouse eggs to generate targeted conditional knockout offspring. Founder animals F0 identified by polymerase chain reaction (PCR) analysis were bred with wild‐type (WT) mice to test germline transmission. The loxP insertion was identified by PCR analysis in F1 and F2 generations. To generate the germ cell‐specific knockout of *Pex5*, *Pex5* ^*flox/flox*^ and *Pex5* ^*flox/+*^ mice were crossed with *Stra8‐Cre* mice. All mice were maintained on the C57BL/6J genetic background.

### 2.2 Genotyping

Genotyping was performed by PCR amplification of genomic DNA extracted from mice tail tips.^24^ The PCR product of the *Pex5* mutant allele was 300 bp. The WT band was 202 bp, and the *Stra8‐Cre* band was 500 bp. The PCR primers are listed in Table S1.

### 2.3 Assessment of fertility

To test fertility, control and conditional knockout males about 2 months old were paired with two random adult WT females. The pregnancy rate and a number of offspring were recorded. The fertility test lasted for at least 3 months.

### 2.4 Antibodies

Primary and secondary antibodies used and their dilutions are listed in Table S2.

### 2.5 Histological, immunohistochemical, and TUNEL assays

Testes and epididymides were fixed in Bouin's solution or 4% paraformaldehyde (PFA) for 12 h at room temperature, embedded in paraffin, and sectioned (4 μm thickness) using routine methods. After deparaffinization and rehydration, tissue sections were stained with haematoxylin and eosin (H&E) or subjected to antigen retrieval for immunohistochemical analysis. After endogenous peroxidase inhibition with 3% H_2_O_2_ for 15 min and blocking with goat or rabbit serum for 15 min at room temperature, the tissue sections were incubated in primary antibodies overnight at 4°C. Staining and chromogenic reactions were conducted with the Streptavidin‐Peroxidase‐Biotin Kit (PV‐9003, ZSGB‐BIO, China) and DAB Kit (ZLI‐9017, ZSGB‐BIO, China) according to the manufacturer's instructions. For immunofluorescence analysis, sections were boiled in sodium citrate buffer (#C1032, Solarbio, China) for 20 min, washed in phosphate‐buffered saline (PBS) three times, and blocked with 5% goat serum for 30 min, and later incubated with primary antibodies overnight at 4°C. Slides were washed and incubated with secondary antibodies for 1 h at 37°C, followed by incubation with 4', 6‐diamidino‐2‐phenylindole. All steps after adding the secondary antibody were performed without exposure to light. Apoptotic cells were analysed by Terminal deoxynucleotidyl transferase‐mediated dUTP‐biotin nick end labeling (TUNEL) assay, which was performed with the TUNEL Kit (#KGA7072, keyGEN BioTECH) following the manufacturer's instructions. Fluorescence images were captured using a fluorescence microscope (OLYMPUS, BX53, Japan).

### 2.6 Meiotic chromosome spread and immunofluorescence staining

Testes from adult mice were subjected to a spermatocyte surface spreading assay as previously reported.^25^ After the tunica albuginea was removed, testicular tubules were pretreated with hypotonic buffer (30 mM Tris pH 7.4, 17 mM trisodium citrate dihydrate, 5 mM ethylenediaminetetraacetic acid [EDTA], 50 mM sucrose, and moderate PBS) for 30 min. Subsequently, short fragments of testicular tubules were suspended in single cells in 100 mM sucrose and treated with 1% (w/v) PFA solution containing 0.15% Triton X‐100 and 1 N NaOH. Cells were spread to a thin cell layer on the slides. Finally, these slides were dried for 3 h at room temperature or overnight at 4°C in a closed box with high humidity. Air‐dried slides were used for immunofluorescence staining. The experimental procedure was roughly the same as the staining method of the tissue section.

### 2.7 Transmission electron microscopy

The testes tissue was rapidly isolated from control and *Pex5* ^*−/−*^ male mice, prefixed with 3% glutaraldehyde, postfixed in 1% osmium tetroxide, dehydrated in an acetone series, infiltrated in Epox 812, and embedded. Semithin sections were stained with methylene blue, and ultrathin sections, obtained with a diamond knife, were stained with uranyl acetate and lead citrate. Sections were examined under a JEM‐1400 Flash transmission electron microscope.

### 2.8 PNA and PAS staining of murine germ cells

Peanut agglutinin (PNA) was used to detect the outer acrosomal membrane of spermatids, and periodic acid‐Schiff (PAS) was used to stain acrosomal sugars. After deparaffinization and rehydration, tissue sections were stained with fluorescein isothiocyanate (FITC)‐conjugated PNA (#L7381, 1:1000, Sigma Aldrich, Germany) for 30 min at 37°C. PAS staining was performed on testes sections with the PAS Stain Kit (#G1280, Solarbio, China) according to the manufacturer's instructions.

### 2.9 RNA‐seq and analysis

Total RNA was isolated from whole testes at 35 days postpartum (P35). RNA‐seq libraries were constructed from 1 μg of RNA using a NEBNext UltraTM RNA Library Prep Kit (NEB, USA). Sequencing was performed on an Illumina NovaSeq 6000 platform with 350‐bp paired‐end reads. Differentially expressed gene (DEG) analysis was performed using the DESeq2 R package. Gene ontology (GO) and Kyoto Encyclopedia of Genes and Genomes (KEGG) enrichment analysis of DEGs was implemented with the clusterProfiler R package. Library construction, sequencing, and data analyses were performed by Annoroad Genomics Co., Ltd. (Beijing, China).

### 2.10 Statistical analysis

All values are presented as mean ± standard deviation (SD). Statistical data were analysed using GraphPad PRISM 8. All experiments included at least three independent samples and were repeated at least three times. The variances of the two groups were compared by the Student's *t*‐test with an unpaired, two‐tailed distribution, and significance was defined as *p < 0.05, **p < 0.01, ***p < 0.001, ****p < 0.0001; n.s., not significant.

## 3 RESULTS

### 3.1 PEX5 was required for spermatogenesis and male fertility in mice

**FIGURE 1:**
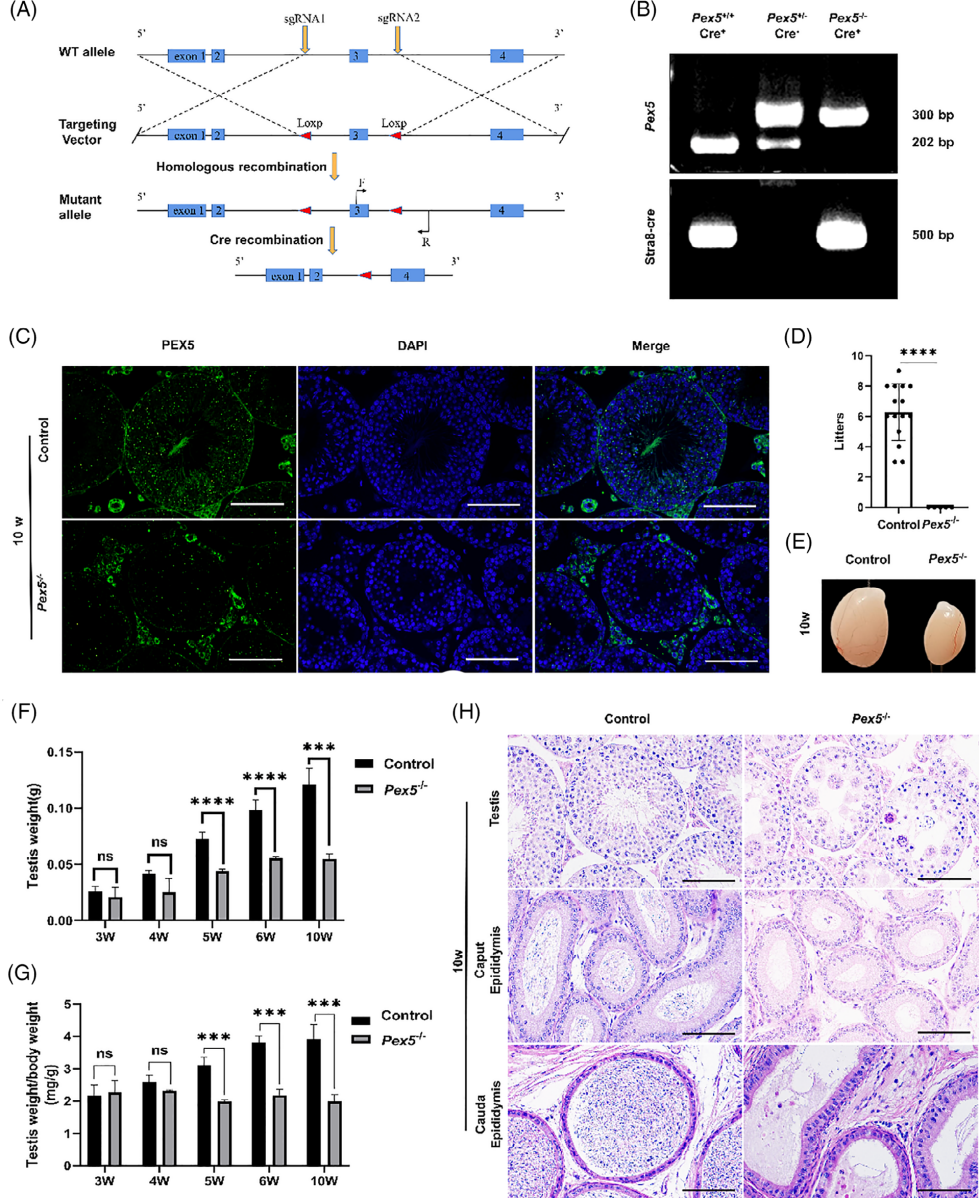
Pre‐meiotic disruption of *Pex5* led to spermatogenetic defects and male infertility. (A) Targeting strategy for the generation of *Pex5* conditional knockout mice. (B) Polymerase chain reaction genotyping in control and *Pex5* ^*−/−*^ mice. (C) Immunofluorescence detection of PEX5 location in mice testes. (D) Fertility test showing sterile *Pex5* ^*−/−*^ male mice. *n* = 5 for each genotype. Error bar, mean ± SD. (E) Determination of testes size in control and *Pex5* ^*−/−*^ mice at 10 weeks. (F) Comparison of testes weight between control and *Pex5* ^*−/−*^ mice at different ages. *n* = 4. Error bar, mean ± SD. (G) Comparison of testes/body weight ratios between control and *Pex5* ^*−/−*^ mice at different ages. *n* = 3. Error bar, mean ± SD. (H) Haematoxylin and eosin (H&E) staining of the testes and epididymides in adult control and *Pex5* ^*−/−*^ mice. Scale bar = 50 μm.

To investigate the role of PEX5 in male reproduction, CRISPR/Cas9 technology, and the Cre/loxP system was used to generate conditional *Pex5*‐knockout germ cells (*Pex5* ^*−/−*^) mice (Figure 1A). The resultant deletion was verified by PCR (Figure 1B). In addition, the knockout of *Pex5* was confirmed using immunofluorescence staining, which revealed that an amount of PEX5 was expressed in the germ cells of the control testes, and the protein levels of PEX5 were significantly reduced in the testes of *Pex5* ^*−/−*^ mice (Figure 1C). PEX5 was significantly downregulated in *Pex5* ^*−/−*^ testes compared with the control as verified by the western blot (Figure S1). On the whole, these results consistently indicated that *Pex5* ^*−/−*^ mice were generated successfully.

Although the *Pex5* ^*−/−*^ male mice matured and copulated normally, they were completely infertile (Figure 1D). The sizes of the testes of pubertal to adult *Pex5* ^*−/−*^ male mice were smaller than those of littermate control mice (Figure S1 and Figure 1E–G). Many apoptotic spermatocytes of the pachytene stage began to emerge from 2 weeks and gradually increased until multinucleated giant cells (MNCs) were formed at 4 weeks (Figure S1). Histological examination showed no elongated spermatids in the seminiferous tubules, and vacuolar degeneration and numerous MNCs formed by round spermatids or spermatocytes in adult *Pex5* ^*−/−*^ testes, which were not observed in the control testes (Figure 1H). Consistent with this, no sperm was found in the caput or cauda epididymis of adult *Pex5* ^*−/−*^ mice (Figure 1H). Therefore, PEX5 was essential for spermatogenesis and male fertility.

### 3.2 *Pex5* deletion impaired spermatocyte meiosis

To determine the mechanism of azoospermatism and sterility in *Pex5* ^*−/−*^ mice, it was necessary to investigate whether proliferation, differentiation of spermatogonia, and meiosis of spermatocytes were normal. The expression of different cell markers was analysed in adult mice (10 weeks). Immunohistochemical staining analysis showed that the number of PLZF‐positive and KIT‐positive cells per tubule of *Pex5* ^*−/−*^ mice, representing undifferentiated and differentiated spermatogonia, respectively, was the same as the control (Figure S2a). Also, immunofluorescence staining of Proliferating cell nuclear antigen revealed a normal presence of proliferation cells in both adult control and *Pex5* ^*−/−*^ mice (Figure S2b). Therefore, spermatogonia proliferation and differentiation in *Pex5* ^*−/−*^ mice were normal.

**FIGURE 2:**
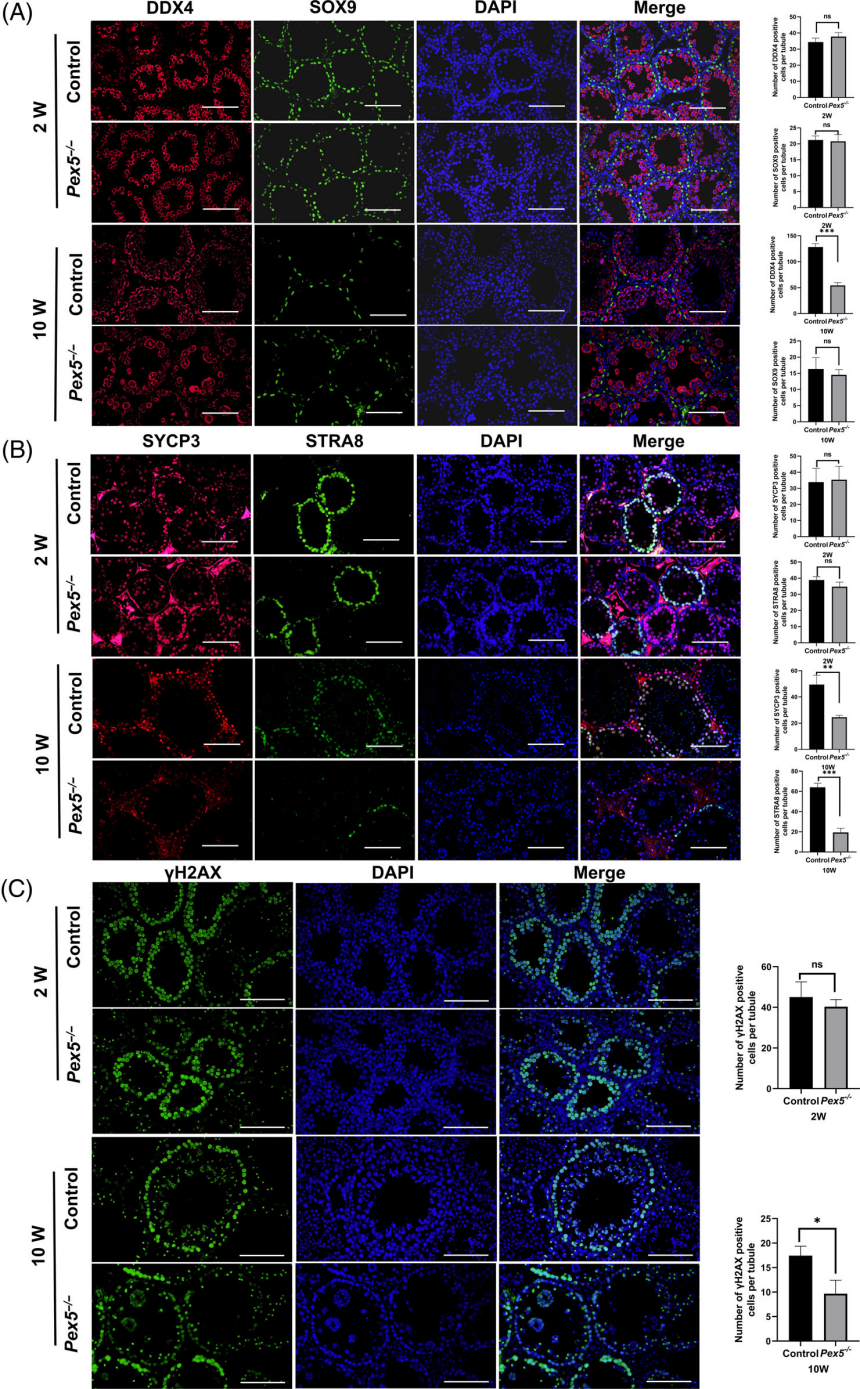
Abnormal meiosis in *Pex5* ^*−/−*^ mice testes. (A) Immunofluorescence staining of DDX4‐positive germ cells and SOX9‐positive Sertoli cells in control and *Pex5* ^*−/−*^ testes at 2 and 10 weeks. Number of DDX4‐positive cells and SOX9‐positive cells per tubule in control and *Pex5* ^*−/−*^ testes. Error bar, mean ± SD. (B) Immunofluorescence staining of STRA8‐positive and SCP3‐positive spermatocytes in control and *Pex5* ^*−/−*^ male mice at 2 and 10 weeks. The number of STRA8‐positive and SCP3‐positive cells per tubule in control and *Pex5* ^*−/−*^ testes. Error bar, mean ± SD. (C) Immunofluorescence staining of γH2AX‐positive cells in control and *Pex5* ^*−/−*^ testes at 2 and 10 weeks. A few cells in MNCs showed positive staining. The number of γH2AX‐positive cells per tubule in control and *Pex5* ^*−/−*^ testes. Error bar, mean ± SD.

The number of germ and Sertoli cells was further investigated by DDX4 and SOX9. There was no significant quantitative change in germ cells or Sertoli cells in the testes of *Pex5* ^*−/−*^ 2‐week‐old mice (Figure 2A). Adult *Pex5* ^*−/−*^ mice (10 weeks) had a normal level of SOX9‐positive Sertoli cells, but the germ cells were reduced (Figure 2A). To determine whether *Pex5* ^*−/−*^ spermatocytes could enter meiosis, 2‐week testicular sections were stained with antibodies to STRA8 and SCP3. According to immunofluorescence staining, the meiotic initiation marked by STRA8 and homologous chromosome synapsis marked by SCP3 were normal in the testes of *Pex5* ^*−/−*^ 2‐week‐old mice (Figure 2B). γH2AX indicated that the germ cells in 2‐week‐old *Pex5* ^*−/−*^ mice had entered meiosis and initiated programmed DNA DSBs (Figure 2C). However, the STRA8, SCP3, and γH2AX positive cells were significantly lower in adult *Pex5* ^*−/−*^ mice testes (10 weeks) than in the control (Figure 2B,C). Most cells were round spermatids in MNCs, and a few cells in MNCs were positive for γ‐H2AX, indicating that a small number of spermatocytes existed in MNCs (Figure 2C). Thus, *Pex5* was required for spermatocyte meiosis in adult mice.

### 3.3 *Pex5* deletion impaired meiotic DSBs repair and potential crossover loci in spermatocytes

**FIGURE 3:**
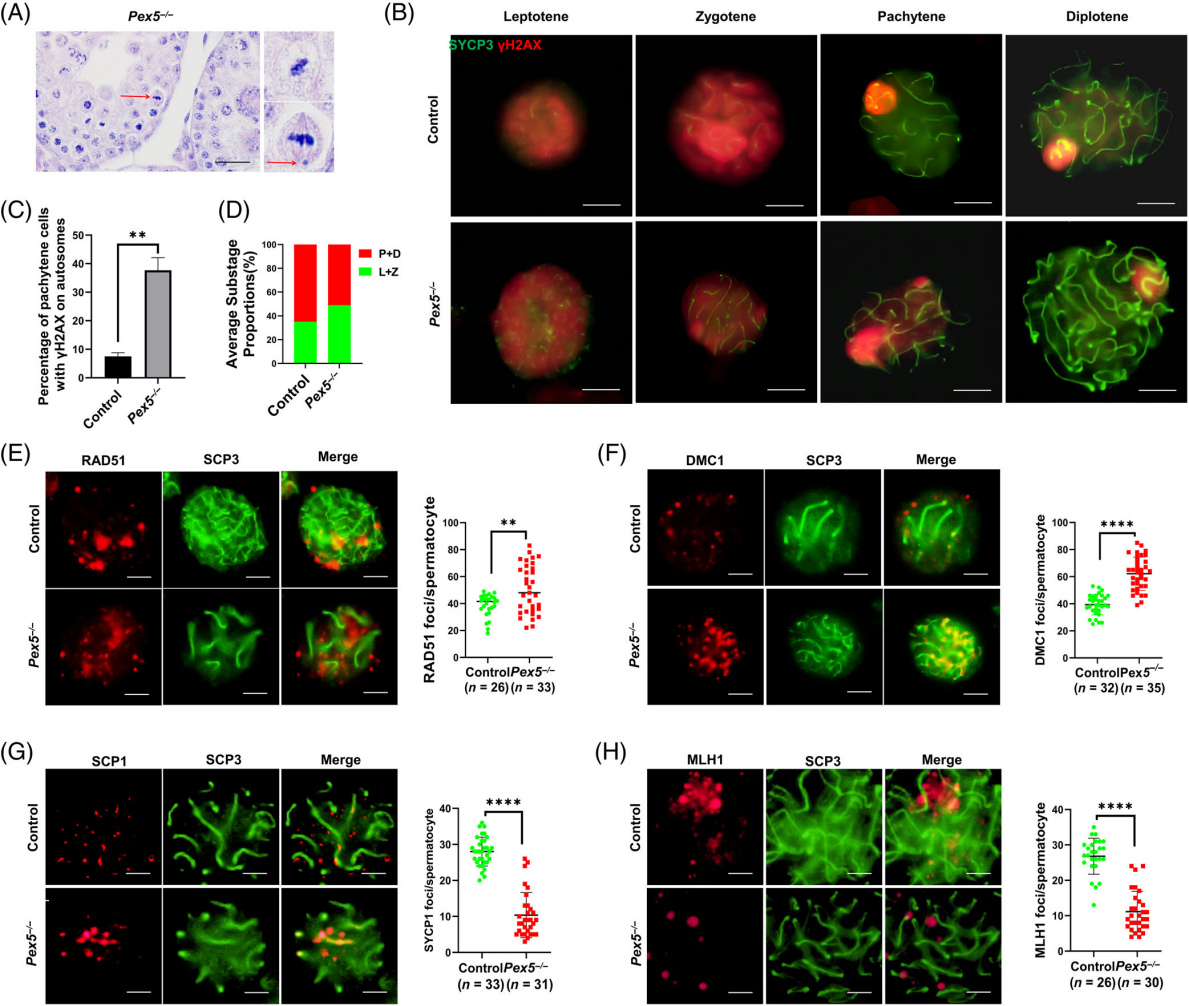
Impact of *Pex5* deletion on DNA double‐strand break repair and crossover formation in spermatocytes. (A) Haematoxylin and eosin staining of sections of paraffin‐embedded testes from *Pex5* ^*−/−*^mice. Arrows indicate univalent chromosome lagging in metaphase cells. (B) Meiotic spermatocyte spread in control and *Pex5* ^*−/−*^ mice at 10 weeks. Co‐staining was performed with SCP3 and γH2AX antibodies. (C) Percentage of pachytene spermatocytes that contained γH2AX foci on their autosomes. (D) Spermatocyte stage proportion in control and *Pex5* ^*−/−*^ mice based on SYCP3 and γH2AX immunofluorescence staining of spermatocyte spreads. L, leptotene; Z, zygotene; P, pachytene; D, diplotene. (E), (F) Immunofluorescence staining of RAD51 and DMC1 on spermatocyte spreads from control and *Pex5* ^*−/−*^ mice. Graphs show the quantification of RAD51 and DMC1 foci numbers per cell at the pachytene stage. The number of analysed spermatocytes (*n*). Error bar, mean ± SD. (G), (H) Immunofluorescence staining of SCP1 and MLH1 on spermatocyte spreads from control and *Pex5* ^*−/−*^ mice. Graphs show the quantification of SCP1 and MLH1 foci numbers per cell at the pachytene stage. The number of analysed spermatocytes (*n*). Error bar, mean ± SD. Scale bar = 10 μm.

Abnormal metaphase cells containing lagging chromosomes were found on H&E‐stained sections of *Pex5* ^*−/−*^ mice (Figure 3A). To further confirm the delayed stages in meiosis, the prophase of meiotic progression by chromosomal spreads from the testes of adult mice was assessed and co‐immunostained with SCP3 and γH2AX (Figure 3B). All stages from leptotene to diplotene were observed in spermatocytes in both control and *Pex5* ^*−/−*^ mice. When DSBs occurred, γH2AX signals were observed throughout the nucleus in leptotene and zygotene (Figure 3B). When the autosomal breaks were repaired, γH2AX signals were restricted to the sex chromosome in pachytene and diplotene in control mice (Figure 3B). Nevertheless, unrepaired DSBs with γH2AX signals partially retained on autosomes occupied 37.7% of *Pex5* ^*−/−*^ pachytene, which was markedly higher than 7.5% of the control (Figure 3C). Another evidence of this impairment was that the proportion of leptotene/zygotene spermatocytes increased in the testes of *Pex5* ^*−/−*^ mice (Figure 3D). Thus, DSB repair and the transition from zygotene to pachytene of spermatocytes were severely impaired in *Pex5* ^*−/−*^ spermatocytes. Therefore, *Pex5* deletion decreased the progression of MI; in other words, *Pex5* ^*−/−*^ spermatocytes were partially arrested at the prophase of MI.

Thus, the different stages of DSB processing in chromosome spreads of pachytene *Pex5* ^*−/−*^ spermatocytes were further observed by co‐immunostaining SCP3 with RAD51 and DMC1. Both RAD51 and DMC1 were significantly accumulated in *Pex5* ^*−/−*^ spermatocytes, compared with the control, indicating incomplete DSB repair (Figure 3E,F). The phenotype of *Pex5* ^*−/−*^ mice was similar to many mutants with defective synapsis or recombination.^26^, ^27^ Therefore, SCP1 and MLH1 were used as markers to examine potential crossover loci in pachytene spermatocytes. Synapsis and crossover recombination significantly decreased in pachytene of *Pex5* ^*−/−*^ spermatocytes compared with the control (Figure 3G,H). Thus, PEX5 was indispensable in meiosis and played a pivotal role in DSB repair and synapsis in spermatocytes.

### 3.4 Abnormal microtubule structure and impaired acrosome development in *Pex5* ^*−/−*^ mice

**FIGURE 4:**
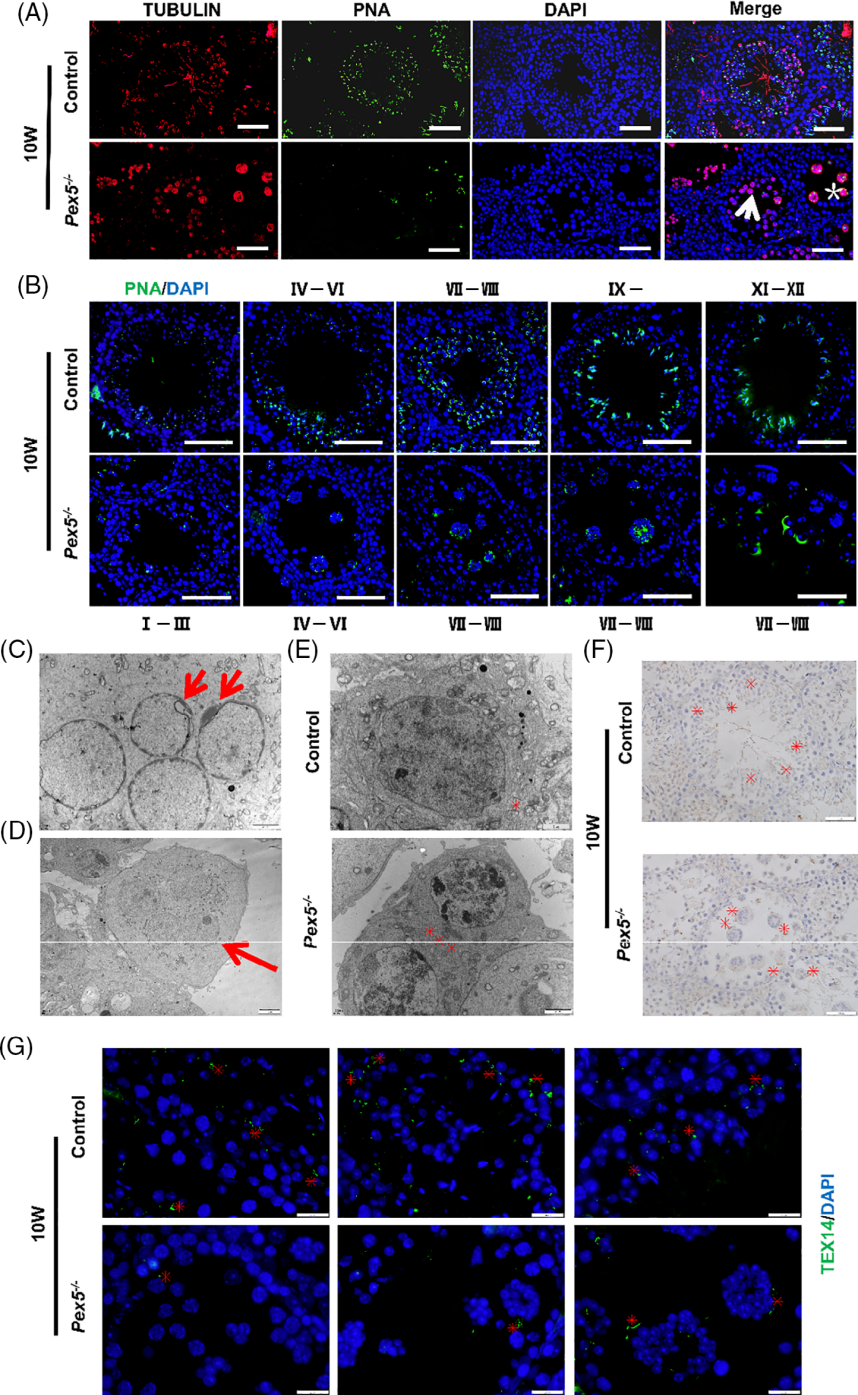
Loss of *Pex5* caused microtubule and acrosome defects during spermatogenesis. (A) Immunofluorescence staining of tubulin and fluorescein isothiocyanate (FITC)‐conjugated PNA in sections of control and *Pex5* ^*−/−*^ mice testes. Most of the cells in multinucleated giant cells (MNCs) were round spermatids. Tubulin was highly expressed in spermatids in the control, but it expressed in MNCs and spermatocytes in *Pex5* ^*−/−*^ mice. The signal of PNA staining was found in spermatids in the control, but it was observed in MNCs and spermatocytes in *Pex5* ^*−/−*^ mice. Compared with controls, the number of PNA‐positive cells was greatly reduced in *Pex5* ^*−/−*^ mice (arrow, spermatocytes; asterisk, MNCs). (B) Immunofluorescence staining of FITC‐conjugated PNA at every stage in control and *Pex5* ^*−/−*^ mice testes. Spermatids had 12 typical forms of acrosomes in the control. The cap acrosome and the crescent acrosome at stages IV–VIII were abnormal and the acrosome was arrested at VII–VIII stage in *Pex5* ^*−/−*^ mice. Scale bar = 50 μm. (C) Abnormal acrosome development of spermatids in MNCs (arrow). Scale bar = 2 μm. (D) Representative transmission electron microscopy (TEM) images of *Pex5* ^*−/−*^ germ cells showing MNCs (arrow). Scale bar = 5 μm. (E) TEM reveals electron dense material lining cytoplasmic channels (intercellular bridges [ICBs]) between germ cells, and the ICB was narrow in control but widened and deformed in *Pex5* ^*−/−*^ (asterisks). Scale bar = 2 μm. (F) Immunohistochemical staining of testes by using the anti‐TEX14 antibody showed ICBs, which were mislocalized in MNCs of *Pex5* ^*−/−*^ (asterisks). Scale bar = 50 μm. (G) Immunofluorescence staining of seminiferous tubules using the TEX14 antibody. Round or C‐shaped structures of ICBs were observed in the control, while they were reduced and deformed in *Pex5* ^*−/−*^ (asterisks), indicating ICB instability. Scale bar = 20 μm.

Next, spermatogenesis was judged by observing microtubule structure and acrosome morphology in *Pex5* ^*−/−*^ mice. PNA and tubulin were used to identify acrosome development and microtubule structure. Immunofluorescence staining showed that tubulin was highly expressed in round spermatids and the tail of elongated spermatids in the control, but it was highly expressed in spermatocytes and aggregated in MNCs in *Pex5* ^*−/−*^ mice (Figure 4A and Figure S3a). Furthermore, the number of acrosome‐positive cells was greatly reduced, and PNA was only detected in MNCs in *Pex5* ^*−/−*^ mice (Figure 4A and Figure S3a). Round spermatids in the control had the three typical forms of acrosomes—punctate Golgi (I–III), cap (IV–VI), and crescent (VII–VIII) (Figure 4B). The acrosome of elongated spermatids in the control was located at the top of the nucleus (IX–XII) (Figure 4B). However, the cap acrosome at stages IV–VI and the crescent acrosome at stages VII–VIII were abnormal in *Pex5* ^*−/−*^ mice (Figure 4B). PAS staining affirmed the presence of less acrosomal positive cells except for MNCs, and the acrosome was fused into clumps in *Pex5* ^*−/−*^ mice (Figure S3b). Transmission electron microscopy (TEM) also showed abnormal acrosomes in MNCs (Figure 4C). Thus, there were many abnormal round spermatids solely in MNCs, while only a few round spermatids and no elongated spermatids were present in *Pex5* ^*−/−*^ mice. Furthermore, the round spermatids are arrested at stages VII–VIII. Defects of the intercellular bridges (ICBs) were observed when MNCs started to form (Figure 4D,E). Normal ICBs were narrow and exhibited a C‐shaped or semicircular profile with membrane‐bound dense material; while two neighbouring cells were connected by widened and deformed ICBs, and the cytoplasmic membrane fused in *Pex5* ^*−/−*^ (Figure 4E). TEX14 is a component of germ cell ICBs. In immunohistochemical staining by using an anti‐TEX14 antibody, a lot of ICB structures were labelled in control testes; while they were observed only in MNCs but were absent from the germ cells in *Pex5* ^*−/−*^ mice (Figure 4F). Tex14‐positive cells in *Pex5* ^*−/−*^ were less than in the control. Mislocalization of ICBs was found in *Pex5* ^*−/−*^ testes (Figure 4G). This suggests that PEX5 knockout caused ICB dysregulation and induced MNCs.

**FIGURE 5:**
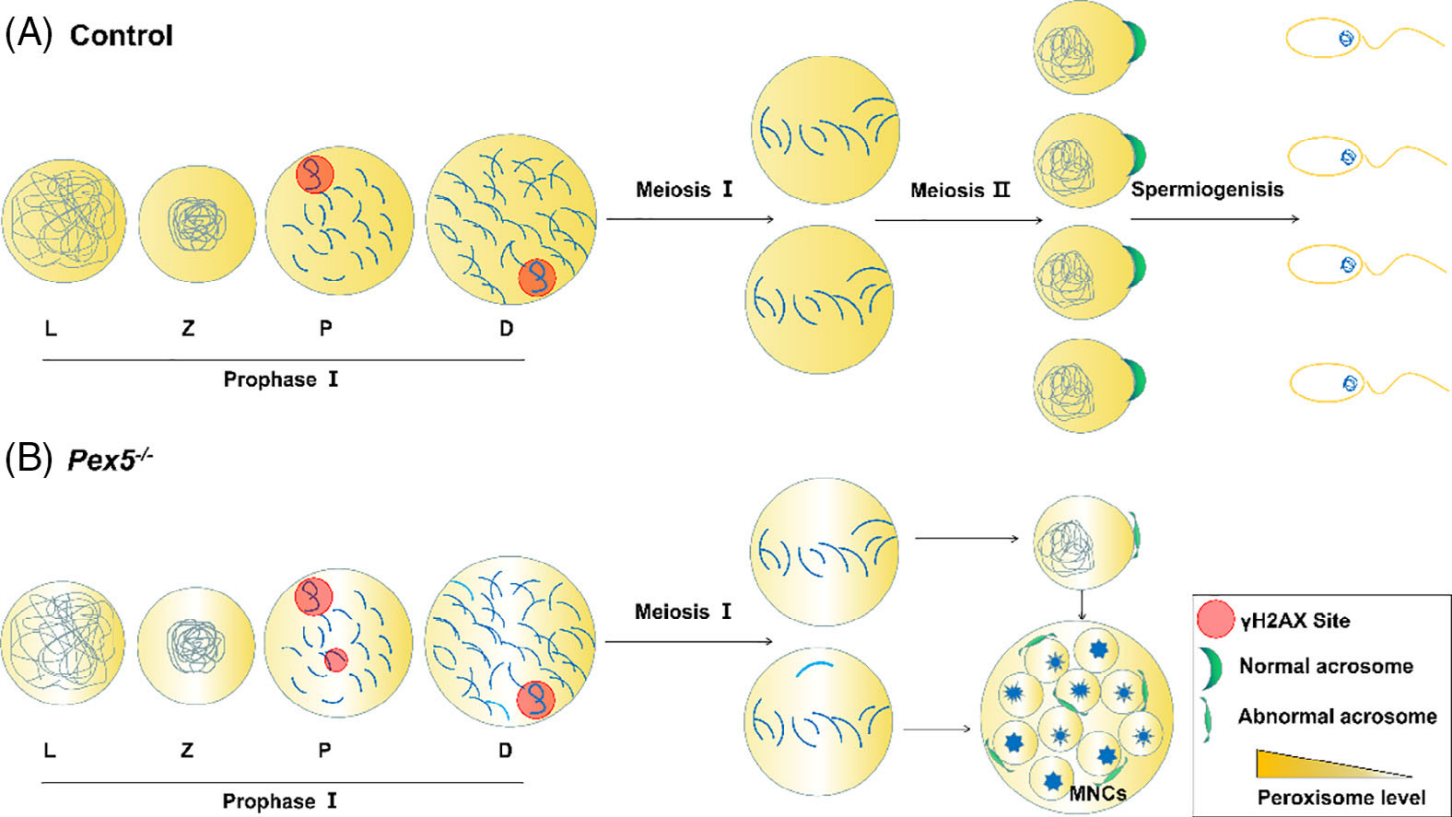
*Pex5* was necessary for spermatogenesis. (A) Schematic showing the function of PEX5 in meiotic prophase as well as (B) the phenotypes of *Pex5* ^*−/−*^ mice. *Pex5* knockout caused delayed double‐strand break repair and homologous recombination or crossover formation (red dots and light blue lines, respectively). Spermatogenesis of *Pex5* deletion was arrested at MII partially, finally forming apoptotic multinucleated giant cells (MNCs). And some germ cells completed meiosis, then the spermatids started to differentiate abnormally in MNCs. L, leptotene; Z, zygotene; P, pachytene; D, diplotene.

In summary, these results indicate that the postnatal deletion of *Pex5* by *Stra8‐Cre* has no influence on the differentiation or proliferation of spermatogonia but plays an important role in meiosis during spermatogenesis. Spermatocytes underwent two meiotic divisions, and round spermatids developed into elongated spermatids in the control (Figure 5A). *Pex5* knockout caused delayed DNA DSBs repair and reduced homologous recombination or crossover formation. Some germ cells failed to complete meiosis and spermatogenesis in *Pex5* ^*−/−*^ mice was arrested partially at meiosis. Although a number of spermatocytes completed meiosis and the spermatids started to differentiate, they initiated false acrosome biogenesis, finally forming MNCs (Figure 5B). These germ cells were eventually cleared during the meiosis or spermatids stage, resulting in spermatogenesis failure and male sterility.

### 3.5 PEX5 maintained peroxisomal function and regulated ROS in germ cells

**FIGURE 6:**
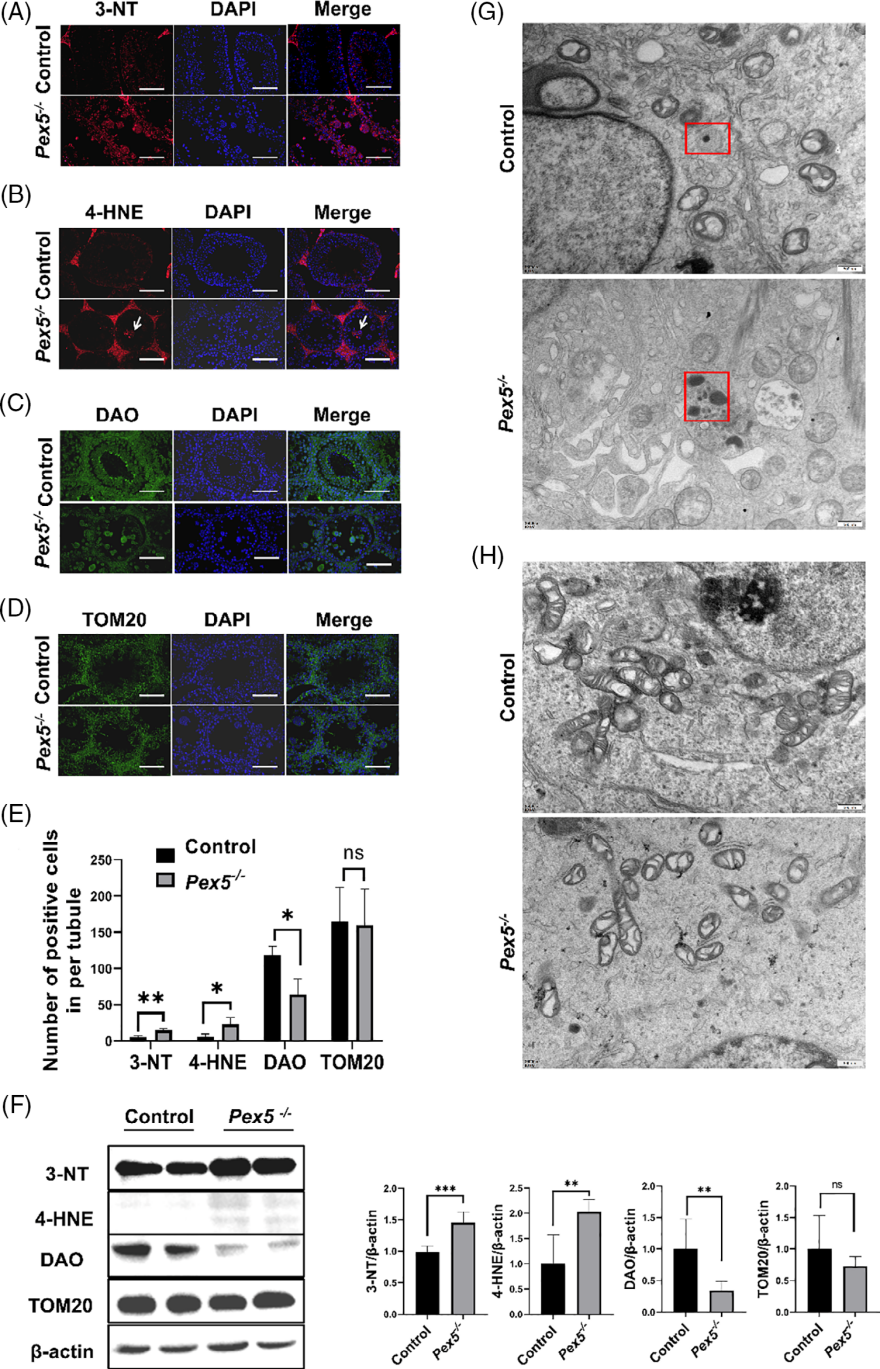
*Pex5* was required for oxidative stress regulated by peroxisomes. (A), (B) Immunofluorescence staining of 3‐NT and 4‐HNE in control and *Pex5* ^*−/−*^ testes (arrows, 4‐HNE‐positive germ cells). (C), (D) Immunohistochemical staining of D‐amino‐acid oxidase (DAO)‐positive peroxisomes and TOM20‐positive mitochondria from control and *Pex5* ^*−/−*^ male mice. Scale bar = 50 μm. (E) The number of 3‐NT, 4‐HNE, DAO, and TOM20‐positive cells per tubule. Error bar, mean ± SD. (F) Western blot was used to detect the protein expression levels of 3‐NT, 4‐HNE, DAO, and TOM20 in the testes of control and *Pex5* ^*−/−*^ mice. (G) Peroxisomal structures in control and *Pex5* ^*−/−*^ spermatocytes, showing multiple crystal nuclei or without membrane in *Pex5* ^*−/−*^ spermatocytes (square frame), as identified by transmission electron microscopy (TEM). Scale bar = 500 nm. (H) Mitochondrial structures in control and *Pex5* ^*−/−*^ spermatocytes identified by TEM. Scale bar = 500 nm.

Peroxisome is a critical organelle for ROS metabolism. As a redox‐regulated import receptor, PEX5 coupled with CAT constitutes a cellular defence mechanism against oxidative stress.^28^ Therefore, the ROS markers 4‐HNE and 3‐NT were examined by immunofluorescence and western blot in adult mice testes. The expression of both 3‐NT and 4‐HNE significantly increased in the germ cells of *Pex5* ^*−/−*^ mice testes compared with the control (Figure 6A,B,E,F). Thus, the loss of PEX5 led to an increase in ROS and induced cellular damage.

Peroxisomes and mitochondria play important roles in oxidative balance. Thus, the focus was on the morphology or structure of peroxisomes and mitochondria in *Pex5* ^*−/−*^ mice testes. D‐amino‐acid oxidase (DAO) and TOM20 were used to evaluate the amounts of peroxisomes and mitochondria, respectively, based on their immunopositive signal.^29^ The peroxisomes decreased and were arranged sparsely in the testes of male *Pex5* ^*−/−*^ mice (Figure 6C,E,F). However, no significant difference in the number of mitochondria was found between control and *Pex5* ^*−/−*^ mice testes (Figure 6D–F). In addition, TEM showed the testicular ultrastructure in both the control and *Pex5* ^*−/−*^ groups. TEM findings showed that PEX5 deficiency could lead to multiple crystal nuclei and peroxisomes with a missing membrane structure in germ cells (Figure 6G). However, mitochondria in control and *Pex5* ^*−/−*^ mice testes showed no significant changes (Figure 6H).

### 3.6 Dysregulated apoptosis and autophagy activity in male *Pex5* ^*−/−*^ mice

**FIGURE 7:**
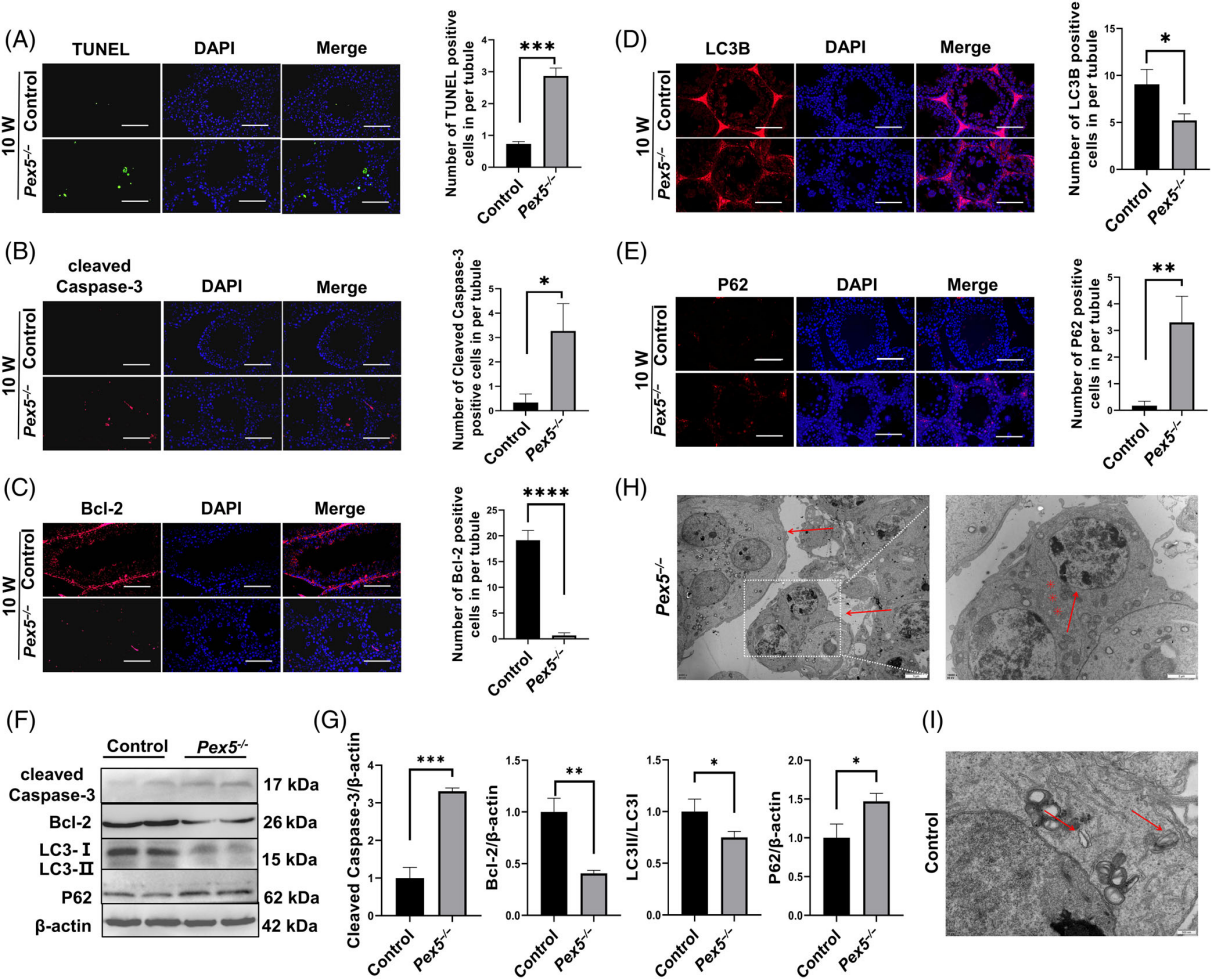
Apoptosis and autophagy activity detected in *Pex5* ^*−/−*^ male mice. TUNEL assays of the testes sections prepared from control and *Pex5* ^*−/−*^ mice at 10 weeks. (A) TUNEL‐positive germ cells are shown in green. Scale bar = 50 μm. Error bar, mean ± SD. (B), (C) Immunofluorescence staining was used to detect cleaved caspase‐3 and Bcl‐2 in the testes of control and *Pex5* ^*−/−*^ mice. Scale bar = 50 μm. (D), (E) Immunofluorescence staining was used to detect LC3B and P62 in the testes of control and *Pex5* ^*−/−*^ mice. Scale bar = 50 μm. (F) Western blot was used to detect the protein expression levels of cleaved caspase‐3, Bcl‐2, LC3, and P62 in the testes of control and *Pex5* ^*−/−*^ mice. (G) Protein expression levels of cleaved caspase‐3, Bcl‐2, LC3II/I, and P62 in the testes of control and *Pex5* ^*−/−*^ mice. (H) Multinucleated giant cells were observed in *Pex5* ^*−/−*^ mice testes under transmission electron microscopy (TEM) (left, arrow). Scale bar = 5 μm. Representative TEM images of *Pex5* ^*−/−*^ spermatocytes showing apoptotic cells with increasing levels of heterochromatin (right). Scale bar = 2 μm. (I) Representative TEM images of autophagic vacuoles in control spermatocytes (arrow). Scale bar = 500 μm.

To study the mechanism of developmental defects in the seminiferous epithelium of *Pex5* ^*−/−*^ mice, apoptosis and autophagy activity was investigated in the testes. Compared with the control mice, the frequency of apoptotic germ cells in seminiferous tubules of *Pex5* ^*−/−*^ mice was higher, as demonstrated by the TUNEL assay (Figure 7A and Figure S4). Immunofluorescence results also showed that the level of cleaved caspase‐3 was elevated, and Bcl‐2 reduced in the testes of *Pex5* ^*−/−*^ mice (Figure 7B,C), both of which were apoptotic markers, further indicating increased apoptotic activity. However, autophagy was suppressed in *Pex5* ^*−/−*^ testes, as indicated by the increased protein levels of P62 and decreased LC3 analysed by immunostaining (Figure 7D,E), which are markers of mammalian autophagy. Western blot showed the same results: the protein levels of cleaved caspase‐3 and P62 increased, and Bcl‐2 and LC3II/LC3I decreased in the testes of *Pex5* ^*−/−*^ mice (Figure 7F,G).

In addition, TEM showed the testicular ultrastructure in control and *Pex5* ^*−/−*^ groups. MNCs were formed close to the seminiferous tubule lumen of *Pex5* ^*−/−*^ mice (Figure 7H). Apoptotic characteristics were observed, which mainly involved chromatin condensation and margination of nuclear chromatin in the testes of *Pex5* ^*−/−*^ mice (Figure 7H). There were fewer autophagosome‐like structures in the spermatogenic cells of the *Pex5* ^*−/−*^ group compared with the control group (Figure 7I). Therefore, *Pex5* deletion elevated apoptosis and decreased autophagy activity in the testes.

### 3.7 RNA‐seq analysis of the testes in control and *Pex5* ^*−/−*^ mice

**FIGURE 8:**
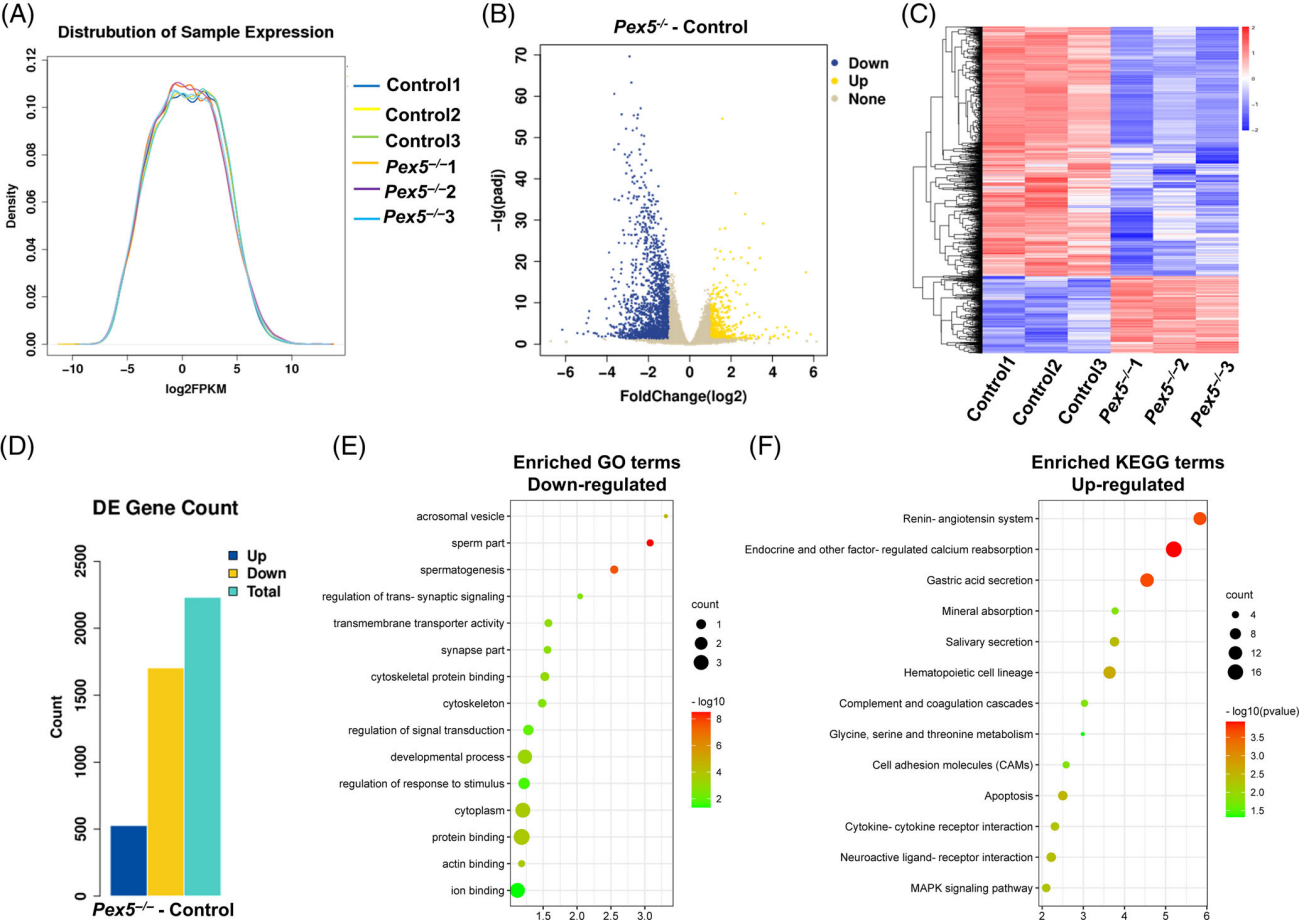
Ablation of *Pex5* altered gene expression in the testes. (A) Distribution of sample expression. (B) Volcano plot showing differential gene expression between control and *Pex5* ^*−/−*^ mice testes at 35 days postpartum (P35). Values are presented as normalized log2(FPKM) (upregulated genes, yellow; downregulated genes, blue). (C) Heatmap of differentially expressed genes (DEGs) between control and *Pex5* ^*−/−*^ mice testes at P35. (D) Upregulated and downregulated genes in *Pex5* ^*−/−*^ mice (cut‐offs: FC >2 and *p* < 0.05). (E) Gene ontology (GO) terms for downregulated DEGs (cut‐offs: FC >2 and *p* < 0.05). (F) Kyoto Encyclopedia of Genes and Genomes (KEGG) terms for upregulated DEGs (cut‐offs: FC >2 and *p* < 0.05).

To further understand the mechanism of PEX5 in spermatogenesis at the molecular level, transcriptome sequence analysis was performed in control and *Pex5* ^*−/−*^ testes of P35 mice. First, the distribution of each sample expression was similar (Figure 8A), indicating that the data was reliable. A set of genes with expression levels substantially altered by the absence of PEX5 was identified (Figure 8B), and more genes were downregulated than upregulated in *Pex5* ^*−/−*^ mice (Figure 8C). Based on RNA‐seq data analysis, 1704 genes were downregulated and 527 genes were upregulated using adjusted *P* < 0.05 (Figure 8D). We checked some ICB‐related genes: TEX14, RBM44, MKLP1, ZO1, TOP2B, KIAA1210, CEP55, et al. However, we did not find any significant changes in the expression of these genes in our RNA‐seq results.

Subsequently, to identify the enriched biological processes of DEGs, GO analysis was performed. Notably, the downregulated genes in *Pex5* ^*−/−*^ testes were significantly enriched in terms related to synapsis, acrosome, cytoskeleton, and development (Figure 8E), which might partially explain the abnormal meiotic arrest with aberrant synapsis, abnormal acrosome, and microtubule development. Moreover, KEGG enrichment analysis was performed to identify the affected biological pathways. A portion of the upregulated genes was associated with apoptosis. Apoptosis was one of the terms of the top five upregulated pathways (Figure 8F). Thus, the transcriptome data suggested that *Pex5* deletion had extensive effects on spermatogenesis, especially meiosis and apoptosis.

## 4 DISCUSSION

**FIGURE 9:**
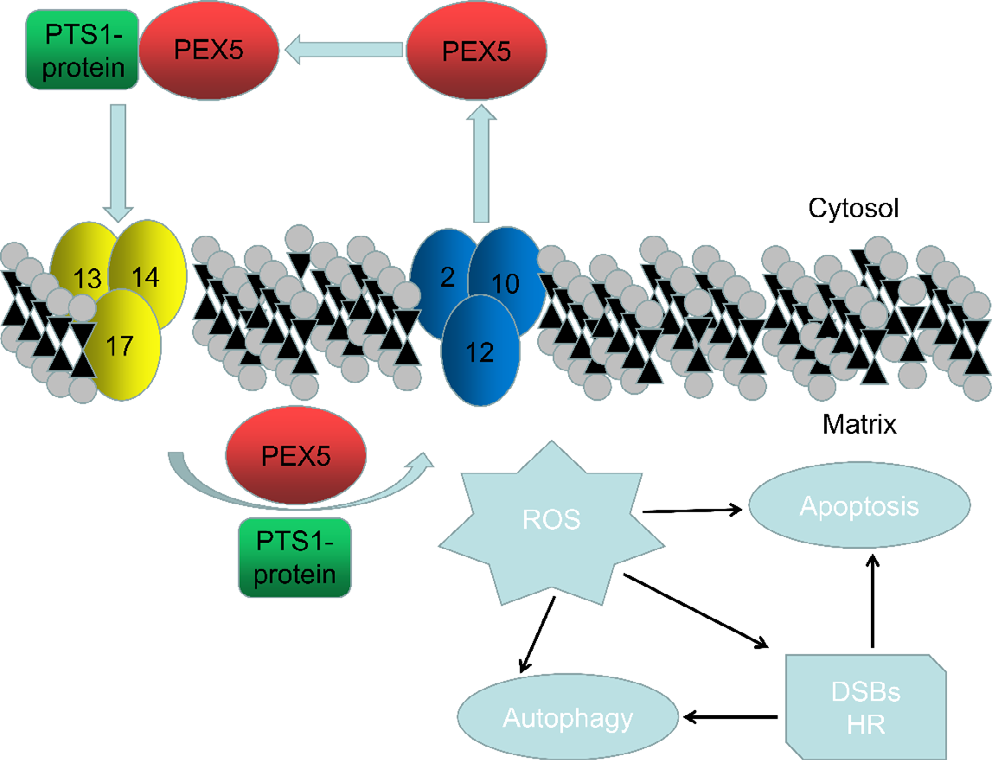
Schematic diagram of peroxisomal biogenesis factor 5 (PEX5) function in the testes. PEX5 was essential for peroxisome formation. It recognized and bound to cytosolic soluble proteins with peroxisomal targeting signal 1 (PTS1), transported them into the peroxisomal matrix, and left the peroxisomal membrane for another round of import. PEX5 regulated reactive oxygen species (ROS) homeostasis, affecting meiosis, apoptosis, and autophagy of spermatocytes. DSB, double‐strand break

PEX5 mediates protein import pathways in human peroxisome biogenesis(Figure 9).^30^ If the import of peroxisomal matrix proteins is compromised, proteins may produce toxic byproducts and engage in metabolic reactions that would be detrimental to cells.^31^ In less severe forms of peroxisomal dysfunction, as described in patients with AMN and X‐ALD, testicular changes, including Leydig cell degeneration, seminiferous tubule reduction, or even spermatogenic arrest, were observed.^32^, ^33^ Disruption of peroxisomal function results in spermatogenesis defects, including spermatocyte cytokinesis failure in *Drosophila* and germ cells interrupted in round spermatids in mice.^34^, ^35^ Nevertheless, whether *Pex5* plays a role in spermatogenesis remains unknown. To explore the function of *Pex5* in male fertility, a conditional *Pex5* knockout mouse model was generated using *Stra8‐Cre*. Male mice with *Pex5* deletion were sterile, the testes' weight was significantly reduced, and sperm were absent in the epididymis. In addition, PEX5 is required for maintaining normal ROS levels and the progression of meiosis during spermatogenesis.

ROS accumulation can impair DNA DSBs repair or homologous recombination.^7^, ^8^, ^36^ During meiosis, DNA DSBs lead to phosphorylation of γH2AX by the activation of ataxia telangiectasia‐mutated (ATM) protein kinase.^24^ Most DNA DSBs are repaired during the zygotene to pachytene stages except for the sex chromosomes, and some DSBs go through crossover resolution.^37^ At the pachytene stage of *Pex5* ^*−/−*^ spermatocytes, the persistence of γH2AX on autosomes suggested that some DSBs were not properly repaired. Moreover, the number of RAD51 and DMC1 foci, which were the distribution of proteins involved in DSB repair, was significantly increased at the pachytene stage in *Pex5* ^*−/−*^ testes compared with the control. This result indicated that DNA DSBs repair was delayed. In mammals, synapsis between homologous chromosomes is essential for the completion of prophase I. When some factors were knocked out, spermatocytes had severe defects in meiotic recombination and synapsis.^38^, ^39^, ^40^, ^41^ This is consistent with the observed results. In the control, the homologous chromosomes synapsed at the pachytene stage. However, in *Pex5* ^*−/−*^ testes, most spermatocytes showed arrested development with no chromosome synapsis as indicated by decreased foci of SCP1 and MLH1. In addition, these recombination and crossover defects could trigger pachytene apoptosis as reported.^42^ In the present study, apoptotic spermatocytes significantly increased in *Pex5* ^*−/−*^ mice. Meiotic defects in *Pex5* ^*−/−*^ mice, accompanied by pachytene arrest, DSB repair delays, and crossover formation failure during prophase I, were associated with the loss of spermatocytes and spermatogenesis failure.

Mitochondria are the main organelles producing ROS, and the disruption of mitochondrial integrity can increase ROS production and release them into the cytoplasm.^43^ However, neither the morphology nor the number of mitochondria was altered. Peroxisomes are also important organelles involved in oxidative balance.^44^ The present study showed that the ROS markers 3‐NT and 4‐HNE increased. Peroxisomes were also significantly reduced and showed abnormal morphology. Thus, the occurrence of excessive ROS is because of peroxisome disorder. The ROS‐degrading enzyme CAT was downregulated in *Pex13*/*Stra8‐Cre* ^*+/−*^ mice,^35^ which is consistent with the present study. ROS can induce cell apoptosis and decrease autophagy,^45^, ^46^ while apoptosis and autophagy play important roles in spermatogenesis.^47^ Inhibited autophagy could increase the percentage of apoptotic cells, and autophagy might prevent cells from apoptotic cell death.^48^ PEX5 is a peroxisomal protein associated with autophagy.^49^ In *Pex5* ^*−/−*^ mice, instead of round and elongated spermatids, MNCs were largely present in the seminiferous tubules. MNCs are the consequence of apoptotic spermatogenic cells, leading to defects in germ cell differentiation.^50^ Moreover, autophagy was repressed in the testes of *Pex5* ^*−/−*^ mice. Thus, the disruption of redox balance induced by aberrant *Pex5* and peroxisomes results in the imbalance between apoptosis and autophagy, ultimately leading to spermatogenesis failure.

Collectively, these results support the notion that the loss of post‐meiotic germ cells in *Pex5* ^*−/−*^ mice is due to meiotic arrest and apoptotic spermatocytes. This result is consistent with the study conducted by Chen et al., who traced the male sterile phenotype of *Drosophila pex2* and *pex10* mutants to abnormal spermatocyte development.^34^ However, the possibility that other mechanisms or pathways in *Pex5* ^*−/−*^ germ cells are sufficient to affect spermatocytes, cannot completely be excluded.

The fusion of germ cells resulting in the formation of MNCs occurs primarily due to the opening and ensuing damage to the ICBs.^51^ ICB was a stable cytoplasmic channel connecting cells. It was found that without ICBs spermatogenesis fails to complete meiosis and results in male infertility.^52^ The protein TEX14 is expressed specifically in germ cells and localizes to the ICB in mice and humans. TEX14 co‐localizes with midbody matrix proteins and converts them into stable ICB components.^53^ In our research, the TEX14 protein was expressed improperly and the ICBs were widened and deformed, explaining the formation of MNCs. However, the relationship between PEX5 and ICBs is still unknown. Thus, further studies are required to elucidate the interactions between PEX5 and spermatogenesis.

## AUTHOR CONTRIBUTIONS

Jiangang Gao and Zongzhuang Wen conceived the project. Min Liu, Shuangyuan Liu, Chenyang Song, Haixia Zhu, and Hui Zhao performed the experiment and wrote the manuscript. Bin Wu and Aizhen Zhang analysed data. All authors read and approved the final manuscript.

## CONFLICT OF INTEREST

The authors declare no conflict of interest regarding the publication of this research.

## Supporting information


**Figure S1.** PEX5 is indispensable for spermatogenesis. (a) Western blot showed the protein level of PEX5 in the testes of control and *Pex5* ^*−/−*^ mice. (b) Images of control and *Pex5* ^*−/−*^ testes at 2 weeks, 3 weeks, 4 weeks, 5 weeks, and during adulthood. (c) H&E staining of control and *Pex5* ^*−/−*^ testes from 2 week to 3 month (arrows, apoptotic spermatocytes; asterisks, MNCs). Scale bar = 50 μm.
**Figure S2.** PEX5 cannot affect the proliferation and differentiation of spermatogonia. (a) Immunohistochemistry staining of PLZF/KIT‐positive spermatogonia in control and *Pex5* ^*−/−*^ male mice. (b) Immunofluorescence staining of PCNA‐positive proliferative germ cells in control and *Pex5* ^*−/−*^ male mice. Scale bar = 50 μm.
**Figure S3.** PEX5 is required for normal acrosomes and microtubules in germ cells. (a) Acrosome and microtubule biogenesis were impaired in 6‐week *Pex5* ^*−/−*^ male mice, which were stained by PNA and tubulin. (b) PAS staining was performed for testis sections from adult control and *Pex5* ^*−/−*^ mice. No spermatids were found in *Pex5* ^*−/−*^ mice. Apoptotic spermatocytes appeared in MNCs. Scale bar = 50 μm.
**Figure S4.** PEX5 deletion results germ cell apoptosis. (a) TUNEL assay showed that apoptotic germ cells of *Pex5* ^*−/−*^ mice at 2 weeks were more than the control. (b) TUNEL assay showed that apoptotic germ cells of *Pex5* ^−/−^ mice at 6 weeks were increased significantly than the control. Scale bar = 50 μm.
**Table S1.** Primer sequences.
**Table S2.** Antibody information.

## Data Availability

The data that support the findings of this study are available from the corresponding author upon reasonable request.
