# Supplementary material for: Pre‐meiotic deletion of PEX5 causes spermatogenesis failure and infertility in mice
Source: Cell Prolif. 2022 Nov 26;56(3):e13365. doi: 10.1111/cpr.13365 (PMC9977671; doi:10.1111/cpr.13365)
Supplement: Supplementary file 1 — Figure S1. PEX5 is indispensable for spermatogenesis. (a) Western blot showed the protein level of PEX5 in the testes of control and Pex5 −/− mice. (b) Images of control and Pex5 −/− testes at 2 weeks, 3 weeks, 4 weeks, 5 weeks, and during adulthood. (c) H&E staining of control and Pex5 −/− testes from 2 week to 3 month (arrows, apoptotic spermatocytes; asterisks, MNCs). Scale bar = 50 μm. Figure S2. PEX5 cannot affect the proliferation and differentiation of spermatogonia. (a) Immunohistochemistry staining of PLZF/KIT‐positive spermatogonia in control and Pex5 −/− male mice. (b) Immunofluorescence staining of PCNA‐positive proliferative germ cells in control and Pex5 −/− male mice. Scale bar = 50 μm. Figure S3. PEX5 is required for normal acrosomes and microtubules in germ cells. (a) Acrosome and microtubule biogenesis were impaired in 6‐week Pex5 −/− male mice, which were stained by PNA and tubulin. (b) PAS staining was performed for testis sections from adult control and Pex5 −/− mice. No spermatids were found in Pex5 −/− mice. Apoptotic spermatocytes appeared in MNCs. Scale bar = 50 μm. Figure S4. PEX5 deletion results germ cell apoptosis. (a) TUNEL assay showed that apoptotic germ cells of Pex5 −/− mice at 2 weeks were more than the control. (b) TUNEL assay showed that apoptotic germ cells of Pex5 −/− mice at 6 weeks were increased significantly than the control. Scale bar = 50 μm. Table S1. Primer sequences. Table S2. Antibody information. [file CPR-56-e13365-s001.docx]

**SUPPLEMENTARY FIGURES LEGENDS**

**Figure S1**

**
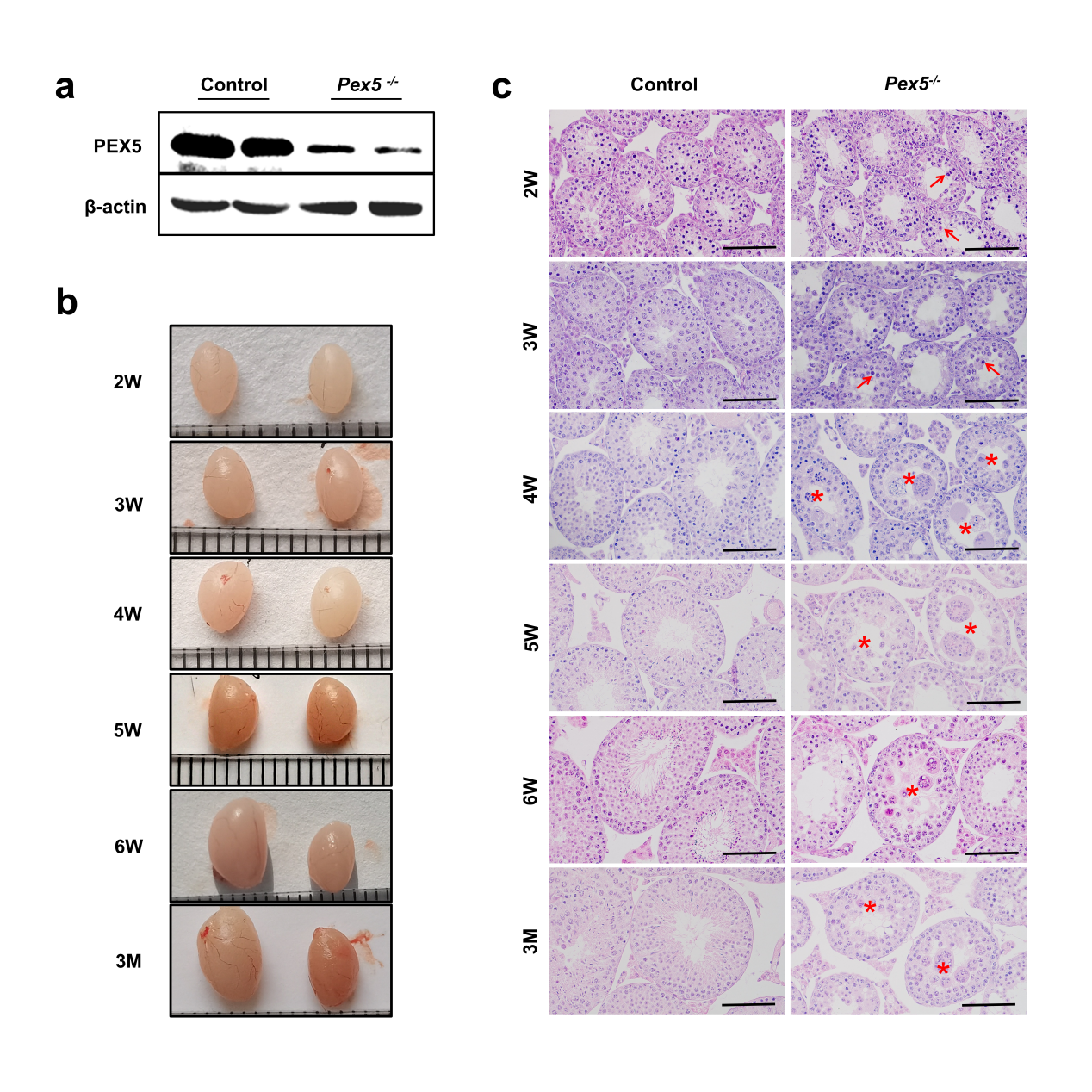
**

**Supplementary Figure S1. PEX5 is indispensable for spermatogenesis.**

1. Western blot showed the protein level of PEX5 in the testes of control and *Pex5^-/-^* mice. (b) Images of control and *Pex5^-/-^* testes at 2-week, 3-weeks, 4-week, 5-week and during adulthood. (c) H&E staining of control and *Pex5^-/-^* testes from 2-week to 3-month (Arrows, apoptotic spermatocytes; Asterisks, MNCs). Scale bar=50 µm.

**Figure S2**

**
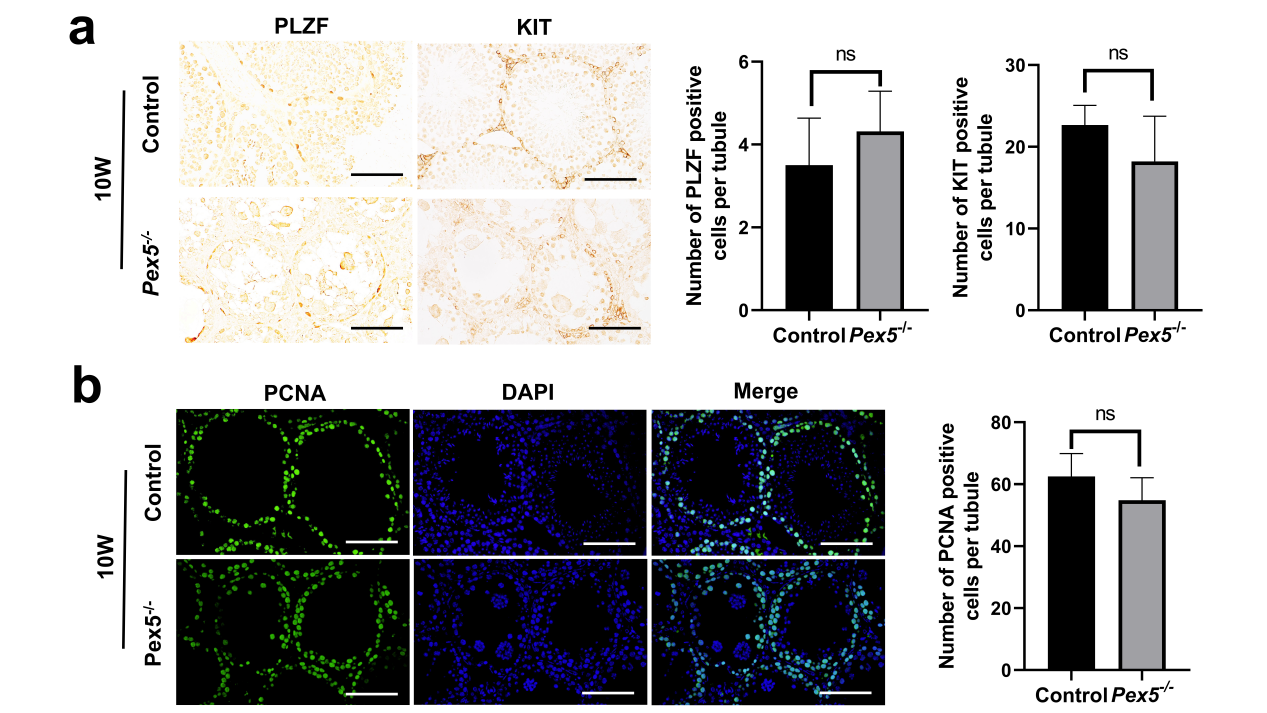
**

**Supplementary Figure S2. PEX5 can’t affect the proliferation and differentiation of spermatogonia**

1. Immunohistochemistry staining of PLZF/ KIT-positive spermatogonia in control and *Pex5^-/-^* male mice. (b) Immunofluorescence staining of PCNA-positive proliferative germ cells in control and *Pex5^-/-^* male mice. Scale bar=50 µm.

**Figure S3**

**
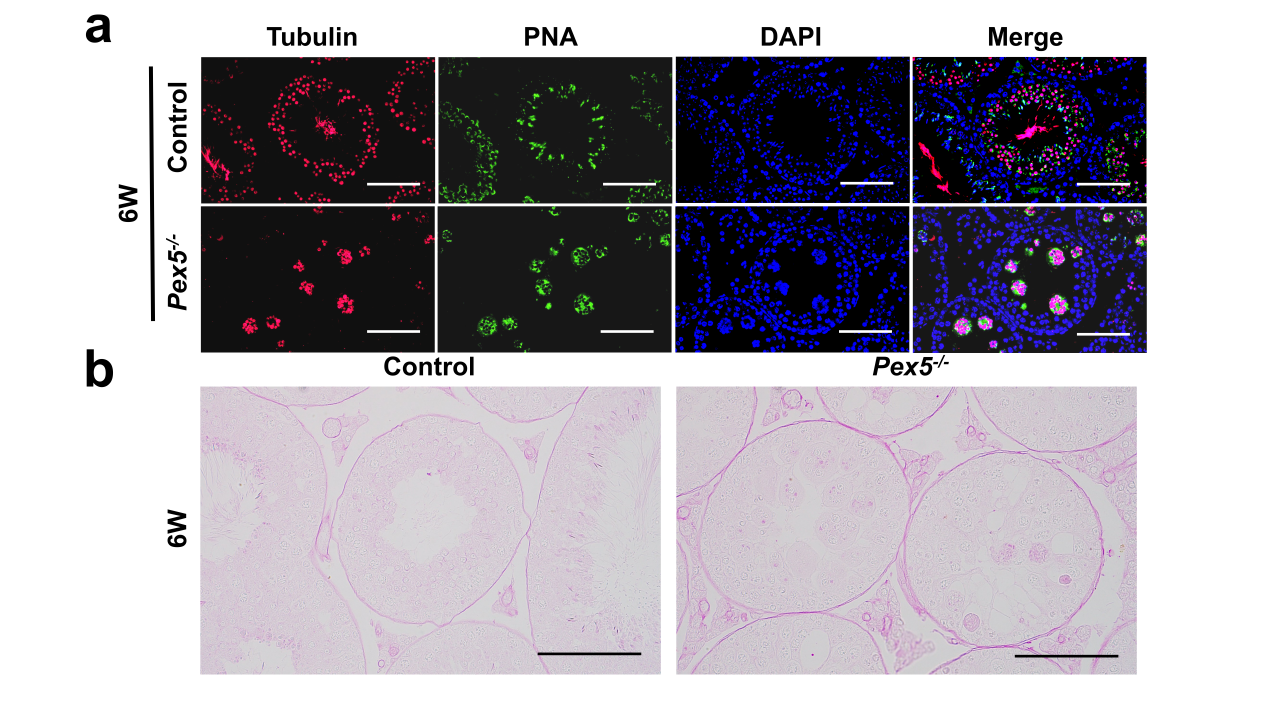
**

**Supplementary Figure S3. PEX5 is required for normal acrosome and microtubule of germ cells.**

1. Acrosome and microtubule biogenesis were impaired in 6-week *Pex5^-/-^* male mice, which were staininged by PNA and Tubulin. (b) PAS staining was performed for testis sections from adult control and *Pex5^-/-^* mice. No spermatids were found in *Pex5^-/-^* mice. Apoptotic spermatocytes appeared in MNCs. Scale bar=50 µm.

**Figure S4**

**
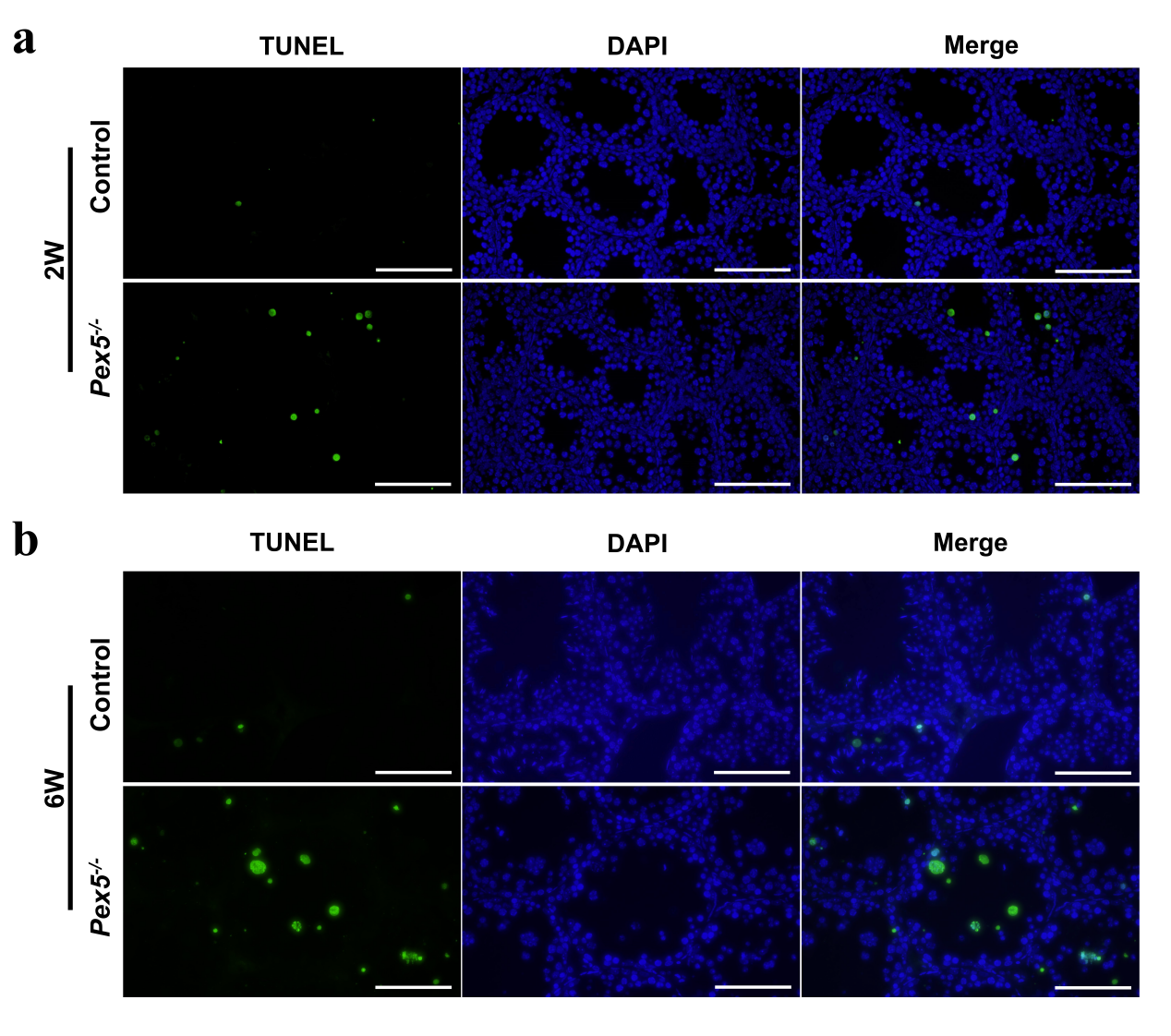
**

**Supplementary Figure S4. PEX5 deletion results germ cells apoptosis.**

1. TUNEL assay showed that apoptotic germ cells of *Pex5*^-/-^ mice at 2-week was more than the control. (b) TUNEL assay showed that apoptotic germ cells of *Pex5*^-/-^ mice at 6-week was increased significantly than the control. Scale bar=50 µm.

**SUPPLEMENTARY TABLES**

**Table S1. Primer sequences.**

| **Genotyping primer** | **The Forward Primer**  **(5'-3' Sequences)** | **The Reverse Primer**  **(5'-3' Sequences)** |
| --- | --- | --- |
| Loxp insertion | GTGGGGAAAGAAGGTGGAAG | CCTGCTTCGCTACTGTTTGG |
| Stra8-Cre | TCGATGCAACGAGTGATGAG | TTCGGCTATACGTAACAGGG |

**Table S2. Antibody information.**

| **Protein name** | **Manufacture**  **(catalogue number)** | **Applications**  **(working dilution)** |
| --- | --- | --- |
| PLZF | RD (#2944) | IHC (1:200) |
| KIT | RD (#1356) | IHC (1:200) |
| PEX5 | Proteintech (#12545-1-AP) | IF, IHC (1:200) Wb(1:1000) |
| SYCP3 | Abcam (ab205846) | IF (1:400) |
| SYCP1 | Abcam (ab15090), | IF (1:100) |
| RAD51 | Abcam (ab176458) | IF (1:400) |
| DMC1 | Proteintech (#13714-1-AP50) | IF (1:400) |
| MLH1 | Proteintech (#11697-1-AP) | IF (1:100) |
| γH2AX | Abcam (ab81299) | IF (1:200) |
| γH2AX | Abcam (ab26350) | IF (1:200) |
| DDX4 | Abcam (ab27591) | IF (1:200) |
| SOX9 | ABclonal (#19710) | IF (1:200) |
| PCNA | SANTA (#sc-56) | IF(1:500) |
| a-tubulin | Sigma (#0000119903) | IF(1:400) |
| 4-HNE | Abcam (ab46545) | IF(1:400) |
| 3-NT | Abcam (ab110282) | IF(1:400) |
| TOM20 | ABclonal (#19403) | IF(1:200) |
| DAO | ABclonal (#5309) | IF(1:200) |
| Cleaved Caspase-3 | CST (#9661) | IF (1:400) Wb(1:1000) |
| Bcl-2 | CST (#3498) | IF (1:400) Wb(1:1000) |
| LC3B | Abcam (ab192890) | IF (1:400) Wb(1:1000) |
| P62 | Abcam (ab109012) | IF (1:400) Wb(1:1000) |
| Fluorescein-Conjugated AffiniPure Goat Anti-mice IgG | ZSGB (#ZF-0312) | IF (1:200) |
| TRITC-Conjugated AffiniPure Goat Anti-mice IgG | ZSGB (#ZF-0313) | IF (1:200) |
| Fluorescein-Conjugated AffiniPure Goat Anti-Rabbit IgG | ZSGB (#ZF-0311) | IF (1:200) |
| TRITC-Conjugated AffiniPure Goat Anti-Rabbit IgG | ZSGB (#ZF-0316) | IF (1:200) |
| Goat Anti-Rabbit IgG (HRPO) | ZSGB (#ZB-2301) | Wb (1:10000) |
| Goat Anti-Mouse IgG (HRPO) | ZSGB (#ZB-2305) | Wb (1:10000) |
